# Hepatic and Splenic Acoustic Radiation Force Impulse Shear Wave Velocity Elastography in Children with Liver Disease Associated with Cystic Fibrosis

**DOI:** 10.1155/2015/517369

**Published:** 2015-11-02

**Authors:** Teresa Cañas, Araceli Maciá, Rosa Ana Muñoz-Codoceo, Teresa Fontanilla, Patricia González-Rios, María Miralles, Gloria Gómez-Mardones

**Affiliations:** ^1^Hospital Universitario Infantil Niño Jesús, 28009 Madrid, Spain; ^2^Universidad Nacional de Educación a Distancia (UNED), 28040 Madrid, Spain; ^3^Hospital Universitario Puerta de Hierro, 28222 Majadahonda, Spain

## Abstract

*Background*. Liver disease associated with cystic fibrosis (CFLD) is the second cause of mortality in these patients. The diagnosis is difficult because none of the available tests are specific enough. Noninvasive elastographic techniques have been proven to be useful to diagnose hepatic fibrosis. Acoustic radiation force impulse (ARFI) imaging is an elastography imaging system. The purpose of the work was to study the utility of liver and spleen ARFI Imaging in the detection of CFLD. *Method*. 72 patients with cystic fibrosis (CF) were studied and received ARFI imaging in the liver and in the spleen. SWV values were compared with the values of 60 healthy controls. *Results*. Comparing the SWV values of CFLD with the control healthy group, values in the right lobe were higher in patients with CFLD. We found a SWV RHL cut-off value to detect CFLD of 1.27 m/s with a sensitivity of 56.5% and a specificity of 90.5%. CF patients were found to have higher SWC spleen values than the control group. *Conclusions*. ARFI shear wave elastography in the right hepatic lobe is a noninvasive technique useful to detect CFLD in our sample of patients. Splenic SWV values are higher in CF patients, without any clinical consequence.

## 1. Introduction

Hepatic chronic disease associated with cystic fibrosis is the second cause of mortality in cystic fibrosis (CF) patients. The real prevalence of cystic fibrosis liver disease (CFLD) is unknown (it is estimated between 13 and 25%) owing to its difficult diagnosis because none of the available tests are specific or sensitive enough and also because there is a discrepancy between the ultrasound findings, the laboratory tests, and the clinical manifestations [1, 2].

CFLD usually appears during childhood, with a peak incidence in teenage years, and usually has a progressive and slow course. Clinical manifestations of the disease are varied and include neonatal cholestasis, asymptomatic hepatomegaly, liver steatosis, biliary tract complications, and portal hypertension, which may require hepatic transplantation [3–5].

CFLD happens more frequently in males, in patients with severe mutations, and in patients with pancreatic insufficiency, with a poor nutritional status, and with a history of neonatal meconium ileus. No specific mutation has been associated with the presence and severity of CFLD [2, 6, 7].

Transaminase levels are altered when the disease is advanced. This is due to the fact that the primary involvement of the disease is biliary, and transaminase values are related to hepatocyte injury rather than to biliary function. For this reason, hepatic involvement is usually subclinical and a combination of clinical examination and different laboratory and imaging techniques is required to make the diagnosis. Dr. Colombo et al. [6] criteria have been accepted for diagnosing CFLD and consist in two of the following findings which are seen at least during two consecutive visits in one year:

1. Hepatomegaly, with liver edge >2 cm below costal margin in the midclavicular line and confirmed by ultrasound,
2. at least 2 of the 3 of AST, ALT, and GGTP above the upper limit of normal.

Ultrasound altered parenchymal pattern (diffuse high echogenicity suggestive of steatosis) is not considered a diagnostic criterium.

The use of hepatic biopsy is controverted in these patients because it is an invasive technique which may have complications and also because there is a 20% of interobserver variability [8]. Furthermore, biliary focal fibrosis, which is the typical pathologic lesion in CFLD, has a patchy liver involvement, which may be related to false negative results after liver biopsy. Because of these reasons biopsy is not used as a screening technique to estimate fibrosis [6, 9, 10], but rather it is used just in dubious cases or when cirrhosis is suspected.

During the last years noninvasive elastographic techniques have been developed and have been proven to be useful to diagnose hepatic fibrosis. Acoustic radiation force impulse (ARFI) imaging (Siemens-ACUSON) is an ultrasound elastographic technique which is integrated in an ultrasound device, based on the measurement of shear wave velocity (SWV) in a ROI (region of interest). SWV is related to mechanic tissue properties; the higher the shear wave speed, the higher the rigidity of the tissue [11].

So far, the authors have found a few articles that report the usefulness of ARFI in the detection of CFLD, in which [9, 12–16] higher SWV values are found in patients with a higher risk of hepatic involvement. However, these studies did not have a group of healthy controls to compare the SWV values. Recently some articles have been published reporting the usefulness of hepatic and splenic ARFI in predicting the risk of portal hypertension and the appearance of oesophageal varices or variceal bleeding in adults [17–20]. However, we have not found studies concerning the usefulness of splenic ARFI in the detection of CFLD.

The aim of the present study is to evaluate the usefulness of hepatic and splenic ARFI in the detection of CFLD.

## 2. Materials and Methods

The study was performed during July 2015. Seventy-two patients with CF disease (45 boys and 27 girls) between 9 months and 18 years old were included. The study was approved by the local ethical committee and written informed consent was obtained in all cases. All patients received the same day abdominal ultrasound, Doppler study, and hepatic and splenic ARFI study and laboratory blood tests within one month. Dr. Colombo et al. criteria were used to diagnose CFLD [6, 9].

SWV values were compared with the values of healthy controls previously studied by the authors [21, 22].

Anthropometric and other data of the patient were saved and studied, including age, gender, body mass index (BMI), history of neonatal meconium ileus, presence of pancreatic insufficiency, treatment with ursodeoxycholic acid, laboratory tests results, and pulmonary function (FEV1). Exclusion criteria were coinfection by hepatotropic viruses, hepatic disease of other causes, and hepatic surgery history. No patient of the selected group was excluded.

### 2.1. Ultrasound

All patients received an ultrasound examination of the abdomen, a portal and splenic color and spectral Doppler examination, and left hepatic vein color and spectral Doppler examination using a convex ultrasound-probe (4C1-probe, Siemens-ACUSON S2000, Mountain View, CA, USA). The abdominal ultrasound included a detailed examination of the liver, and hepatic involvement was graded according to Williams scale [23], Table 1. This scale considers hepatic echotexture, periportal echogenicity, and hepatic surface nodularity and scores the findings, 3 normal, over 4 suggestive of hepatopathy, and 9 suggestive of cirrhosis. Signs of portal hypertension, presence of ascites, and focal liver lesions were evaluated.

### 2.2. ARFI

**Figure 1:**
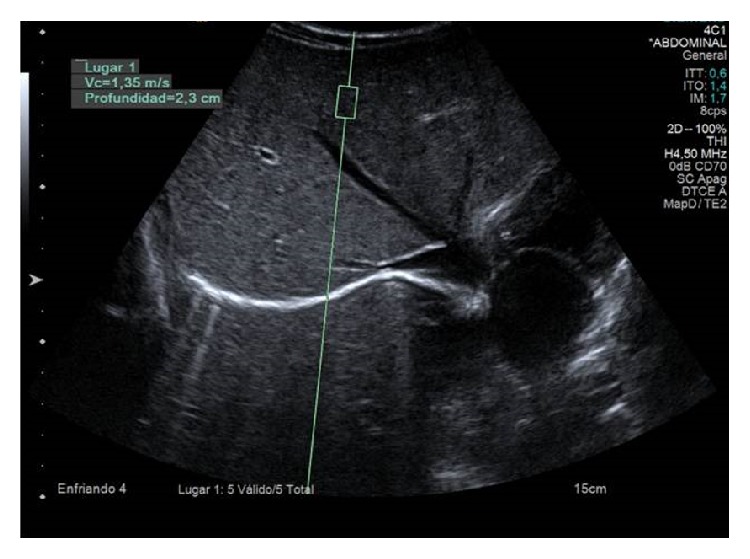
Shear wave velocity (SWV) measurement with the 4 C1 probe in a region of interest 1 × 0.5 cm at a depth of 2,3 cm. SWV = 1.35 m/s.

ARFI hepatic and splenic elastography was performed using a convex multifrequency probe (4C1) on an ACUSON S2000 device (Siemens Medical Solutions, Mountain View, CA, USA) with the specific software for generating and tracking shear waves Virtual Touch Tissue Quantification. All studies were performed by a radiologist with 3 years of experience in performing ARFI. SWV was measured in several ROIs within the liver and the spleen. A rectangular ROI with fixed dimensions (1 × 0.5 cm) was adjusted under ultrasound control in the liver and in the spleen to avoid identifiable blood vessels and biliary structures (Figure 1).

Patients were supine and breathing normally when measurements were performed. For children younger than 7 years old, a subcostal approach was used; in children over 7 years old, an intercostal approach was used. Minimal scanning pressure was applied by the operator. Five valid measurements of SWV were performed in each hepatic lobe (right hepatic lobe (RHL) and left hepatic lobe (LHL)) deeper than 1 cm from the probe; and five valid measurements were performed in the spleen. SWV means and standard deviations were calculated for each hepatic lobe and for the spleen in each patient; the results were expressed in meters per second (m/s). Unreliable velocity measurements caused the machine to automatically display XXXX and were not taken into consideration in shear wave velocity calculations. The number of nonvalid measurements was not registered. Measurements were repeated until five reliable values for a complete examination were obtained. The range of depths between which the measurements were taken was 2,55–5,86 cm in the RHL, 1,96–5,40 cm in the LHL, and 1,72–4,73 cm in the spleen.

Liver and spleen SWV values were compared to those obtained and published by the authors in healthy children [21, 22], Table 2.

### 2.3. Laboratory Tests

Blood tests were performed to all patients either the same day of the ultrasound and ARFI or after one month. The tests included determination of necrobiosis enzymes, alanine-aminotransferase (ALT or GPT) and aspartate-aminotransferase (AST or GOT); of cholestasis enzymes *γ*-glutamyl-transpeptidase, alkaline phosphatase (AP), and total and direct and indirect bilirubin; of hepatic synthesis markers: glucose, albumin, and cholinesterase; and also of prothrombin time.

### 2.4. Statistical Analysis

SPSS 21.0 (IBM, Armonk, NY) was used for statistical analysis. *P* values < 0.05 were considered to indicate statistical significance. Normal distribution was tested using Kolmogorov-Smirnov test. Data followed normal distribution and parametric test was used. To make comparison between two groups Student's *t*-test was used. To make comparison across groups ANOVA test and Bonferroni test were used. Correlations were assessed by Pearson's correlation coefficient. Moreover the areas under the ROC (AUROC) curves were calculated. Cut-off values for the prediction CFLD were defined using Youden's index. The optimal cut-off was defined as the cut-off with the highest sum of sensitivity and specificity.

## 3. Results 

Seventy-two patients with CF were studied. Patient characteristics are shown in Table 3. Fifty-four patients with CF without LD (75%) and 20 with CFLD (86,95%) had pancreatic insufficiency. Ten patients with CF without LD (13,88%) and five with CFLD (21,73%) had a history of meconium ileum. Pulmonary function mean (FEV1) in CFLD patients was 91% and that of patients with CF without CFLD was 87,85%.

### 3.1. Ultrasound

Based on Williams ultrasound score patients were divided into score 3 (normal ultrasound) and scores 4–9 (mild to moderate hepatopathy), Table 2. In the group of patients with CFLD there were 12 patients with normal ultrasound and 11 patients with altered ultrasound. In the group of patients without liver involvement 39 of the patients had a normal ultrasound and 10 patients had altered ultrasound. None of the patients had cirrhosis ultrasound criteria or ascites.

### 3.2. Hepatic ARFI

No statistically significant difference was found between both SWV in the right hepatic lobe and SWV in left hepatic lobe between healthy children (*n* = 60) and the global CF group (*n* = 72) (RHL *P* = 0.386 and LHL *P* = 0.578). In CF patients SWV values were higher in the LHL than in the RHL ($\overset{-}{x}=1.29$ m/s and $\overset{-}{x}=1.22$ m/s, resp., *P* = 0.019), as happens in healthy children [12, 21].

Results of the ANOVA test are shown in Table 4. Comparing the SWV values of RHL in the three groups (group I: healthy children; group II: children with CF without liver disease; group III: children with CFLD) we found a significant value of *F* (*P* = 0.003). The post hoc comparisons indicate that the differences are due to the comparison between group III (CFLD) and groups I (control healthy group) and II (CF without liver disease) because values in the RHL were higher in patients with CFLD. However, no difference was found comparing LHL values (*P* = 0.397).

**Figure 2:**
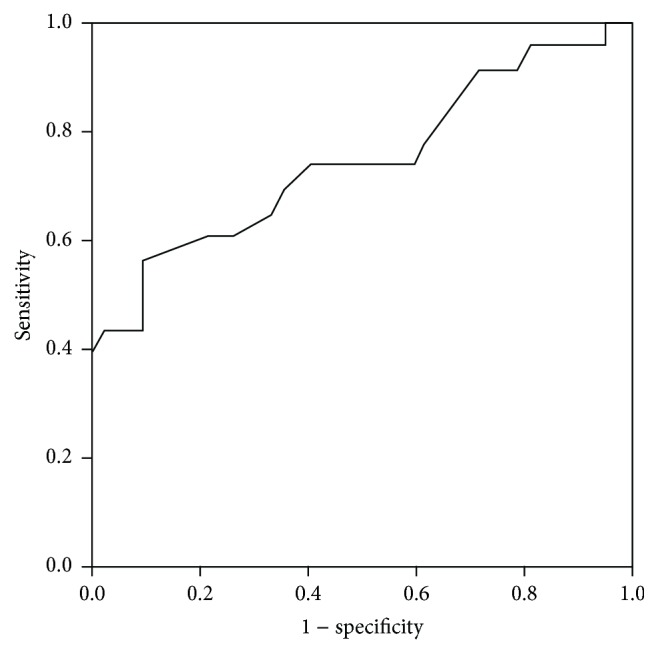
ROC curve for SWV in the RHL.

When we calculate the area under the ROC curve, we found a SWV RHL cut-off value to detect CFLD of 1.27 m/s, with a sensitivity of 56,5% and a specificity of 90,5% (Figure 2). The AUROC for SWV in the RHL was 0.746 *P* < 0.001 (95% CI 0.61–0.88). The AUROC curve (0.529) for SWV in the LHL was not significant.

### 3.3. Splenic ARFI

CF patients were found to have higher SWV than the control group of healthy children (*P* < 0.0001). No significant difference was found when comparing patients with CFLD (*n* = 23) with those CF patients without hepatic involvement (*n* = 49), Table 4.

### 3.4. Laboratory Tests

Pearson correlation was calculated between RHL SWV and the following variables: BMI, FEV 1, GOT, GPT, GGT, FA, bilirubin, glucose, cholinesterase, and prothrombin time. A negative correlation was found with the BMI (−0.239, *P* = 0.044) and a positive correlation was found with GOT (0.397, *P* = 0.001) and GGT (0.386, *P* = 0.001). No other significant correlation was found. Pearson correlation was calculated between splenic SWV and the same variables, and no significant correlation was found.

No significant differences were found in the laboratory variables, BMI and FEV1, between patients with CFLD and patients with CF without CFLD, Table 4.

## 4. Discussion

Owing to the increasing life expectancy of CF patients, CFLD has more time to appear and progress and it is nowadays the second cause of death in CF patients. The mechanisms involved in the pathogenesis of liver disease in CF are largely unknown. The absence or dysfunction of the fibrosis regulator protein (CFTR) is thought to be key in the pathogenetic sequence of cystic fibrosis associated liver disease. CFTR is expressed in the membrane of the intrahepatic and extrahepatic biliary canaliculi cells and in the gallbladder epithelial cells, but it is not present in the hepatocytes, so the damage in this condition is primary biliary and causes increased bile viscosity. This leads to bile duct plugging, and biliary obstruction and chronic cholestasis ensue. Associated pathological findings include inflammation, paraductal mucinous cysts, mural fibrosis, biliary ductal proliferation, and periportal fibrosis [2, 24]. Secondary hepatocyte injury eventually occurs.

**Figure 3:**
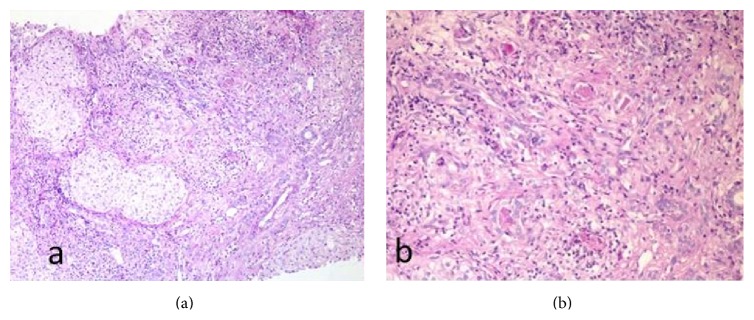
(a) Hepatic parenchyma altered structure: an expanded portal area is shown, with fibrous septa and hepatocytes enclosed in them. Inflammatory cells and ductular proliferation are seen, with positive Periodic Acid Schiff (PAS) content inside several proliferated biliary ducts. (b) Close-up view of the portal area in which four biliary ducts are seen (3 in the top half of the image and one in the lower half of the image) with PAS positive plugging inside. Images lent by Dr. Daniel Azorín, pathology department.

The histologic typical lesion is focal biliary fibrosis (Figure 3). Lesions may be confluent and eventually progress to multilobular cirrhosis. In it, lobules are not equally involved and relatively normal lobules can be found among involved ones. The left hepatic lobe may be more involved than the right one [2]. CFLD diagnosis is difficult and the use of hepatic biopsy is controverted, so noninvasive techniques are being investigated and used, among them elastographic techniques.

ARFI is an elastography imaging system based on SWV measurement that has been found to be a reliable tool for estimating fibrosis in adults, with good accuracy in the diagnosis of significant liver fibrosis and excellent accuracy in the diagnosis of significant fibrosis and cirrhosis [11, 25]. Most of the published articles are based on findings in HCV patients, or else mixed groups of patients including HCV and HBV [26, 27]. Articles based on studies on children are scarce, and in them the sample of patients usually is heterogeneous with different causes of liver disease [28–30]. Since SWV values may be different in different liver diseases, it seems reasonable to study SWV in each disease separately. The main purpose of this study was to find SWV values in CFLD in children and to compare them with CF without liver disease, using as reference of normality the results of previous study of the authors in healthy children [21, 22]. The range of normal values in healthy children was 1.15–1.23 m/s. The results of the present study obtain a cut-off value of 1.27 m/s to detect CFLD, with a sensibility of 56,5% and a specificity of 90,5%. Friedrich-Rust et al. [16] propose a cut value of 1.42 m/s in adult patients with CF with a sensibility of 54.17% and a specificity of 93.90%. The higher cut value obtained by Friedrich may be related to the fact that the studied patients were adult, possibly with more advanced liver involvement. A value of 1.27 m/s really is not very far from a value of 1.23 m/s, with the first considered the cut value for abnormality and the second considered the upper range limit for the mean of SWV in healthy children. This may pose a problem for result interpretation. Studies with larger samples may adjust the values further. In this sense, it would be of interest to carry out a longitudinal study of the values in CF with the same patients. It would be interesting to find if SWV values change along time in CFLD. Presumably SWV values will increase with time in CFLD and in those patients in which LD develops, but this has to be studied specifically.

This study was designed to measure SWV in both hepatic lobes separately. On one hand ARFI systematic study has been agreed in the literature to be performed in the RHL because SWV LHL values are higher than RHL values both in healthy adults and children; in adults LHL values are also more disperse. It is thought that this difference may be due to the closeness of the heart and its pressure on the liver [31, 32]. On the other hand, liver involvement is patchy in CF patients and it has been reported that the LHL may be more affected than the RHL. So another purpose of this work was to study if there are differences between SWV values in both hepatic lobes in CF patients. Our results show that LHL SWV are higher in CF patients, but we have not found difference between patients with and patients without CFLD. So in the same way as Friedrich-Rust et al. [16] we do not think it necessary to measure LHL SWV in the evaluation of liver involvement in CFLD.

Necrobiosis enzymes (AST and ALT) and cholestasis enzymes (GGT and FA) allow detecting hepatic involvement which is not evident at exploration and even with ultrasound. On the other hand, there may be hepatic involvement with normal enzymes. In this study a significant positive correlation has been found between RHL SWV and GOT and GPT values. This association between high transaminase levels and high SWV values has been described in the literature [33] and indeed it is considered a confounder in the estimation of hepatic fibrosis in chronic liver disease in adults, so the recommendation in adults is to perform the evaluation for fibrosis estimation avoiding periods with transaminase flares. It seems reasonable to think that this must be considered also when evaluating CFLD.

Another aim of the present study was to evaluate splenic SWV, taking into account the hemodynamic relationship between liver and spleen, and the secondary splenic involvement of the spleen in chronic liver disease. Considering the spleen, there are a few published articles that report about the higher spleen SWV values and the usefulness of this finding to predict the risk of portal hypertension and the presence of varices and of variceal bleeding in adults [17–20]. In our study splenic SWV values were found to be significantly higher in CF patients than in the control group. However, this finding did not have any clinical consequence or association, there was no plaquetopenia or more frequent infections, and there were no splenomegaly and no ultrasound or Doppler findings suggestive of portal hypertension. Without having histologic confirmation and without any reference in the literature of splenic involvement in CF, it is not possible to ascertain the nature of the splenic high SWV; this could be due to a direct involvement of the disease, but further study of the splenic SWV in these patients is needed.

This study has some limitations; the main one is that there has not been comparison of the diagnosis of hepatic involvement with a gold standard such as the histologic analysis after biopsy. However, the use of an invasive test which is so far not included as a routine procedure in the management or follow-up of CF patients is not justifiable. Also, the healthy reference group was studied by four authors of these articles (Teresa Cañas, Araceli Maciá, Teresa Fontanilla, and María Miralles) in another hospital, though with the same device and convex probe and including children of all ages, so the authors think that those values can still be used as a reference of normality in this study.

The main conclusion of this study is that ARFI shear wave elastography in the RHL was useful to detect CFLD in our sample of patients, and thus it has the potential to be a useful tool in the followup of CFLD patients as a noninvasive technique to evaluate liver involvement and disease progression. Another conclusion is that splenic SWV values are higher in CF patients, without any clinical consequence or association. Further study of splenic SWV is needed.

## Figures and Tables

**Table 1:** Williams ultrasound score [23].

Points	1	2	3

Parenchyma	Normal	Coarse	Irregular

Echogenicity (periportal fibrosis)	None	Moderate	Severe

Liver edge	Smooth	Irregular	Nodular

Score	3 (normal)	>4 (hepatopathy)	9 (cirrhosis)

**Table 2:** Liver and spleen ARFI shear wave velocity normal values [21, 22].

	Mean (SD)	95% confidence interval
SWV right hepatic lobe (m/s)	1.19 (0.13)	1.15–1.23
SWV left hepatic lobe (m/s)	1.27 (0.19)	1.22–1.32
SWV spleen (m/s)	2.17 (0.35)	2.08–2.26

**Table 3:** Patient characteristics.

	Patients without CFLD	Patients with CFLD	All patients with CF
*N*	49	23	72
Sex: H–M	28–21	17–6	45–27
Age: mean (SD) (95% CI)	9,99 (5,3); 0,9–18	10,56 (4,41); 0,9–18	10,18 (5,02); 0,9–18
BMI: mean (SD); 95% CI	17,67 (2,92); 9,5–23	17,3 (3,2); 12,8–26,5	17,56 (2,99); 9,5–26,5
Patients with pancreatic insufficiency	34	20	54
Patients with history of neonatal meconium ileus	5	5	10
Pulmonary function: FEV1: mean (SD)	87,85 (15,21)	91,04 (16,17)	88,96 (15,5)
Williams ultrasound score			
(i) Points <3	39	12	51
(ii) Points between 4 and 8	10	11	21

**Table 4:** Comparison between healthy patients and patients with CF with or without CFLD. ANOVA test and post hoc (Bonferroni) for SWV in right hepatic lobe (RHL), left hepatic lobe (LHL), and spleen. Student's *t*-test for the main variables, BMI and FEV1.

	Group I	Group II	Group III	*F*/*t*	*P*	Post hoc comparison
	Healthy children	Patients with CF without liver disease	Patients with CFLD			
	*N* = 60	*N* = 49	*N* = 23			
	Mean (SD)	Mean (SD)	Mean (SD)			
RHL	1.19 (0.13)	1.18 (0.18)	1.31 (0.16)	6.023	**0.003**	Group I–group III
						Group II-group III
LHL	1.27 (0.19)	1.27 (0.24)	1.34 (0.30)	0.930	0.397	
Spleen	2.16 (0.35)	2.51 (0.30)	2.51 (0.31)	18.154	**<0.0001**	Group I-group II
						Group I–group III
FEV1		87.85 (15.21)	91.05 (16.17)	−0.777	0.440	
BMI		17.67 (2.92)	17.30 (3.19)	0.48	0.633	
GOT		33.94 (13.03)	64.17 (114.47)	−1.82	0.073	
GPT		27.5 (14.08)	54.13 (101.43)	−1.79	0.077	
GGT		15.5 (14.56)	79.79 (272.87)	−1.64	0.106	
AP		187.89 (61.79)	189.65 (73.82)	−0.105	0.917	
TB		0.63 (0.54)	0.31 (0.16)	1.229	0.223	
Glu		93.79 (10.37)	95.26 (16.75)	−0.454	0.651	
Albu		4.03 (0.30)	3.97 (0.27)	0.943	0.349	
Colin		9370.23 (2051.30)	8493.68 (1398.98)	1.693	0.096	

RHL = right hepatic lobe; LHL = left hepatic lobe; FEV1 = pulmonary function; BMI = body mass index; GOT = aspartate-aminotransferase; GPT = alanine-aminotransferase; GGT = *γ*-glutamyl-transpeptidase; AP = alkaline phosphatase; TB = total bilirubin; Glu = glucose; Albu = albumin; Colin = cholinesterase.
